# Loneliness, hopelessness and suicide in later life: a case–control psychological autopsy study in rural China

**DOI:** 10.1017/S2045796020000335

**Published:** 2020-04-24

**Authors:** Lu Niu, Cunxian Jia, Zhenyu Ma, Guojun Wang, Bin Sun, Dexing Zhang, Liang Zhou

**Affiliations:** 1 Department of Social Psychiatry, the Affiliated Brain Hospital of Guangzhou Medical University (Guangzhou Huiai Hospital), Guangzhou, China; 2 Xiangya School of Public Health, Central South University, Changsha, China; 3 School of Public Health, Shandong University, Jinan, China; 4 School of Public Health, Guangxi Medical University, Nanning, China; 5 Shenzhen Kangning Hospital, Shenzhen, China; 6 JC School of Public Health and Primary Care, The Chinese University of Hong Kong, Hong Kong, China

**Keywords:** Loneliness, older adults, psychological autopsy, suicide

## Abstract

**Aims:**

Loneliness is increasingly recognised as a serious public health issue worldwide. However, there is scarce research addressing the association between loneliness and suicide in older adults in rural China. We set out to examine loneliness and other psychosocial factors in elderly suicide cases and explore their interaction effects.

**Methods:**

Using a 1 : 1 matched case–control design, data were collected from 242 elderly suicide cases and 242 living community controls by psychological autopsy method in rural China, including demographic characteristics, loneliness, depression, hopelessness and social support. The chi-square automatic interaction detection (CHAID) tree model and multivariable logistic regression analysis were used to explore the relationships of these factors and suicide.

**Results:**

The CHAID tree model showed that loneliness, hopelessness and depressive symptoms were closely associated with completed suicide and that loneliness and hopelessness interacted with each other. The result of multivariable logistic regression showed that individuals who were unemployed [odds ratio (OR) = 2.344; 95% confidence interval (CI): 1.233–4.457], living alone (OR = 2.176; 95% CI: 1.113–4.254), had lower levels of subjective social support (OR = 2.185; 95% CI: 1.243–3.843), experienced depressive symptoms (OR = 6.700; 95% CI: 3.405–13.182), showed higher levels of hopelessness (OR = 7.253; 95% CI: 3.764–13.974) and felt higher levels of hopelessness × higher levels of loneliness (OR = 2.446; 95% CI: 1.089–5.492) were significantly associated with an elevated suicide risk in older people in rural China.

**Conclusions:**

Regular evaluation of loneliness, hopelessness and depression can help detect older adults who are at risk of committing suicide. Interventions should target social support systems, particularly among people living alone, to alleviate feelings of loneliness and hopelessness. Treating depression is also key to preventing suicide among elderly people in rural China.

## Introduction

Despite the sharp drop in the national suicide rate in China over the past two decades, suicide among older people is of increasing concern (Jiang *et al*., 2018). In 2016, the suicide rate among people over 65 was estimated to be 6.5-fold higher than that of younger people, and it was significantly higher in rural older people than those living in an urban area (Zhong *et al*., 2016). Given the rapid ageing of the Chinese population, the prevention of late-life suicide in rural areas should be a greater priority (Zhang *et al*., 2017). There is a critical need to identify suicide risk factors in this vulnerable population to inform efforts to illuminate potential points of intervention.

Loneliness is an unpleasant feeling due to a mismatch between the level of social connectedness that an individual desires and what he or she has (Russell *et al*., 1980). Increasingly, loneliness is recognised as a serious public health issue worldwide (Cacioppo and Cacioppo, 2018). According to the latest national report of the United Kingdom (UK), 14% of British citizens suffer from loneliness (Mead, 2018). Among older people, the prevalence of loneliness is higher, ranging between 28 and 63% in high-income countries (Mead, 2018; Pimlott, 2018). Loneliness in older people in China might be even more prevalent (Pimlott, 2018). A national report on the living status of elderly Chinese people showed over half of rural older adults are experiencing empty nest syndrome or have been left behind by their adult children (Cai, 2018). Changes in family structure and the social culture of filial piety have weakened adult children's sense of obligation to provide family support to older people (Zhong *et al*., 2018). Consequently, there is a feeling of loneliness among older people in rural China, in particular those who feel left behind (He and Ye, 2014; Wang *et al*., 2017).

Studies have linked loneliness to various correlations of suicidal behaviours, such as depression and hopelessness (Singh and Misra, 2009; Ahmed *et al*., 2014). Loneliness has also been found as a core theme in suicide notes (Synnott *et al*., 2017). Evidence from systematic reviews consistently showed that older people experiencing loneliness tend to have an elevated suicide risk (Madeleine Mellqvist *et al*., 2012; Chang *et al*., 2018). Though loneliness has been found to be associated with suicidal ideation and behaviours, there is a lack of evidence of the role of loneliness in completed suicide in older people in rural China.

Psychological autopsy (PA) is a widely used method to explore the risk factors of completed suicide and has been validated previously in China (Zhang *et al*., 2003; Conner *et al*., 2012). Using a 1 : 1 matched case–control design, this PA study aims to shed light on this research gap. In the current study, we investigated loneliness and other psychosocial factors in elderly suicide cases and matched living controls to explore the interaction effects between loneliness and other factors on suicide in elderly people in rural China.

## Methods

### Study sites

Study sites were selected using a multi-stage stratified cluster sampling strategy. The first stage of sampling involved the selection of provinces. According to the gross domestic product per capita of 31 provinces in mainland China, Shandong province, Hunan province and Guangxi province were chosen from the rank 1–10, 11–20 and 21–31, respectively. The second stage of the sampling design was the selection of rural counties from the selected provinces. In each selected province, counties were stratified into three levels based on per capita income. One county in each stratum from Shandong and Hunan, and two counties in each stratum from Guangxi (as the population sizes of the counties in Guangxi were smaller than those in the other two) were selected using a simple random sampling performed by a computer algorithm. A total of 12 counties from the three selected provinces were included in this study.

### Suicide cases

The study was conducted from June 2014 to September 2015. In each county, suicide cases of individuals aged 60 and above were collected consecutively based on the death certification system. A senior suicidologist briefly trained all village doctors and local public health workers involved in death certification on how to determine a suicide death. They were required to report all elderly suicide deaths, as well as possible cases that they could not determine, to the local Centers for Disease Control and Prevention. Trained investigators finally determined the manner of death after all available, relevant information had been collected.

### Living controls

One person was randomly chosen as a living control from the same village for each suicide case, with the same gender and birth year (±3 years). Whenever a suicide case was identified, the investigators would list and enumerate all elderly persons matching gender and birth year in the same village. One living comparison was then selected from the list randomly using a computer program. If no appropriate control individuals were available in the same village, the investigators expanded the search to the closest villages.

### Procedure of interview

For each suicide case and living control, we selected two informants. Generally, the first informant was a next of kin who lived with the suicide case or the living control. The second informant was a friend, neighbour or remote relative. Interviews with the informants of suicide cases were scheduled 2 to 6 months after the death, while interviews with informants of living comparisons were conducted as soon as the participants and their informants were identified. Each informant was interviewed separately by one fieldworker, and the interview lasted 90 min on average. The details of the subjects' selection and interview procedures have been described in previous publications (Niu *et al*., 2018; Zhou *et al*., 2019).

### Ethics

The authors assert that all procedures contributing to this work comply with the ethical standards of the relevant national and institutional committees on human experimentation and the Helsinki Declaration of 1975, as revised in 2008. All procedures involving human subjects were approved by the institutional review boards of the three universities in the three selected provinces. Written informed consent was obtained from participants in the living control group and all the informants of suicide cases and living comparisons.

### Measures

#### Socio-demographics

We collected socio-demographic information, including gender, age, education level, marital status, employment and living arrangement. We defined participants as left-behind if they had no offspring, or all of their adult offspring lived outside the original township for at least 10 months and visited their parents less than twice in the past year (Zhou *et al*., 2019).

#### University of California Los Angeles Loneliness Scale-6 (ULS-6)

We used the Chinese version of the ULS-6 to measure loneliness (Niu *et al*., 2018). It consists of six items, and each item is rated on a 4-point scale ranging from 1 (never) to 4 (often feel this way). A total score ranges from 6 to 24, and a higher score represents a higher level of loneliness. Good reliability and validity have been demonstrated in the ULS-6 (Niu *et al*., 2018).

#### Geriatric Depression Scale (GDS-30)

We used the GDS-30 to assess depression symptoms in the past week (He *et al*., 2008). Higher scores indicate higher levels of depression. A cut-off score of 10 and 20 represents mild and severe levels of depressive symptoms, respectively. The GDS-30 has shown adequate properties in older Chinese people (He *et al*., 2008).

#### Beck's Hopelessness Scale (BHS-4)

We used the BHS-4 to measure hopelessness (Yip and Cheung, 2006). It consists of four items relevant to success, dark future, breaks and faith. Each item is rated on a 5-point scale ranging from 1 (strongly agree) to 5 (strongly disagree). A total score range from 4 to 20, and a higher score represents a higher level of hopelessness (Yip and Cheung, 2006).

#### Duke Social Support Index (DSSI)

We used the 23-item DSSI to assess social support, which consists of three subscales: social interaction, subjective social support and instrumental social support (Koenig *et al*., 1993). A total score can range from 11 to 45, with higher scores indicating higher levels of social support. The 23-item DSSI has shown good reliability and validity in Chinese older people (Mao *et al*., 2015).

### Data combination

We combined the information provided by the two informants as the proxy data for each suicide case and living control. Regarding socio-demographic data, we relied on the information provided by the first informant. For psychosocial factors, we used answers that were hypothetically associated with elevated risk of suicide from each item of ULS-6, BHS-4, GDS-30 and DSSI, considering any targeting symptoms or behaviours might exist as long as any informant may have observed (Zhang *et al*., 2018; Zhou *et al*., 2019). Thus, positive answer of an item of GDS-30 was used when one of the two informants reported positive; similarly, the higher score of ULS-6 and BHS-4, and lower score of DSSI were used.

### Statistical analyses

Descriptive analysis, pair-sample *t*-tests and two-related-samples Wilcoxon tests were used to describe and compare the socio-demographics, loneliness, depressive symptoms, hopelessness and social support of suicide cases and living controls.

Then, chi-square automatic interaction detection (CHAID) (Kass, 1980), a classification tree model, was applied to identify major factors associated with completed suicide and analyse potential interactions between these factors. Using the significance of a statistical test as a criterion, CHAID evaluates all the values of a potential predictor variable. It merges values judged to be statistically similar to the target variable and maintains dissimilar values. It chooses the most significant variable from the first branch in the decision tree. This process continues recursively until the tree is fully grown (Amirabadizadeh *et al*., 2018). The method allows: (1) identifying complex interactions between variables across the measurement space; (2) identifying the most significant explanatory variable and (3) merging the categories of nominal variables and categorising continuous variables without the loss of information (Emina *et al*., 2013).

In this study, suicide cases were assigned a value of ‘1’, and living controls were assigned a value of ‘0’. The variables, marital status, employment, living alone, being left behind, loneliness, social interaction, subjective social support, instrumental social support, depressive symptoms and hopelessness, were included in the CHAID model. Continuous variables (loneliness, social support and hopelessness) were divided into categorical variables based on the mean scores among the overall participants. Depression was divided into two groups (with or without), using the cut-off value of 10. The CHAID model selected variables with close associations with completed suicide from all influencing factors and visually displayed their interactions in the form of a tree diagram. The effect of the classification tree-based model was evaluated according to the overall accuracy of the predicted categories.

The CHAID model provides a method to explore major factors and their interactions associated with an outcome, whereas logistic regression allows the joint effects of multiple characteristics to be assessed coinstantaneous (Pretorius *et al*., 2007). Thus, all factors (marital status, employment, living alone, being left behind, loneliness, social interaction, subjective social support, instrumental social support, depressive symptoms and hopelessness), as well as the interactions indicated by the CHAID model, were included in the multivariable logistic regression analysis (backward stepwise). All analyses were performed with SPSS version 23.0 (SPSS Inc., Chicago, IL, USA). Significance levels of this study were set at 0.05.

## Results

### Characteristics of suicide cases and living controls

We collected data from 242 suicide cases and 242 living controls. Among the suicide cases, 43.8% were female, and the mean age was 74.4 ± 8.2 years. Among the living controls, 43.8% were female, and the mean age was 74.1 ± 8.2 years. As presented in Table 1, compared with the living controls, the suicide cases tended to have unstable marital status, be unemployed, live alone and be left behind (all *p* values <0.05). Suicides cases reported higher levels of loneliness, hopelessness and depression, as well as lower levels of social support, than the living comparisons (all *p* values <0.01).

**Table 1.** Characteristics of suicide cases and living controls

Variables	Suicide cases (*n* = 242)	Living controls (*n* = 242)	*Z*/*t*	*p*
Gender			–	–
Male	135 (55.8)	135 (55.8)		
Female	107 (44.2)	107 (44.2)		
Age groups (years)			0.667	0.505
60–69	73 (30.2)	70 (28.9)		
70–79	100 (41.3)	110 (45.5)		
⩾80	69 (28.5)	62 (25.6)		
Education			1.557	0.119
Below primary school	111 (45.9)	96 (39.7)		
Primary school	105 (43.4)	116 (47.9)		
Above primary school	26 (10.7)	30 (12.4)		
Marital status			4.707	<0.001
Stable	122 (50.4)	170 (70.2)		
Unstable	120 (49.6)	72 (29.8)		
Employment			2.357	0.018
Employed	41 (16.9)	61 (25.2)		
Unemployed	194 (80.2)	167 (69.0)		
Retired	7 (2.9)	14 (5.8)		
Living alone			3.263	0.001
Yes	64 (26.4)	35 (14.5)		
No	178 (73.6)	207 (85.5)		
Being left-behind			2.263	0.024
Yes	41 (16.9)	25 (10.3)		
No	201 (83.1)	217 (89.7)		
Loneliness [mean (s.d.)]	15.6 (5.0)	11.0 (4.0)	11.854	<0.001
Social support [mean (s.d.)]	22.9 (5.9)	27.5 (6.8)	8.843	<0.001
Social interaction	5.4 (1.3)	6.4 (1.6)	7.616	<0.001
Subjective social support	9.1 (2.9)	11.6 (3.9)	8.075	<0.001
Instrumental social support	8.4 (3.9)	9.5 (3.4)	3.150	0.002
Depressive symptoms [mean (s.d.)]	21.4 (5.9)	9.2 (6.4)	21.604	<0.001
Without (<10)	14 (5.8)	148 (61.2)	11.245	<0.001
With (⩾10)	228 (94.2)	94 (38.8)		
Hopelessness [mean (s.d.)]	14.6 (2.5)	9.7 (2.7)	20.049	<0.001

### Analysis of the classification tree model

**Fig. 1.**
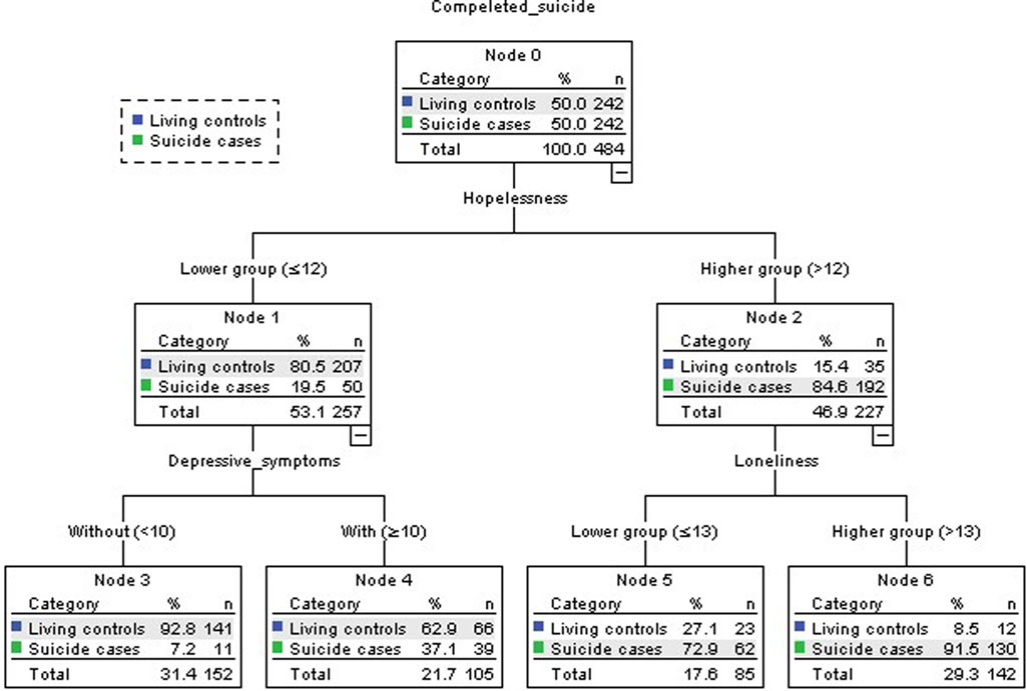
The classification tree model for completed suicide among elderly people in rural China.

A shown in Fig. 1, the classification tree model had two layers of six nodes, including four terminal nodes. The results showed that three variables had close associations with completed suicide among older adults in rural China: hopelessness, loneliness and depressive symptoms. Hopelessness and loneliness interacted with each other.

For the classification tree model, the estimated risk was 0.176, and the standard error was 0.017. The model could accurately estimate 79.3% of suicide cases and 85.5% of living controls. The overall accuracy was 82.4%.

### Multivariable logistic regression for completed suicide

Multivariable logistic regression (backward stepwise) was used to determine factors (marital status, employment, living alone, being left behind, loneliness, social interaction, subjective social support, instrumental social support, depressive symptoms, hopelessness and hopelessness × loneliness) of completed suicide in older adults in rural China. As shown in Table 2, six variables entered the final model: unemployment [odds ratio (OR) = 2.344; 95% confidence interval (CI): 1.233–4.457], living alone (OR = 2.176; 95% CI: 1.113–4.254), lower levels of subjective social support (OR = 2.185; 95% CI: 1.243–3.843), depressive symptoms (OR = 6.700; 95% CI: 3.405–13.182), higher levels of hopelessness (OR = 7.253; 95% CI: 3.764–13.974) and higher levels of hopelessness × higher levels of loneliness (OR = 2.446; 95% CI: 1.089–5.492).

**Table 2.** Multivariate logistic regression for completed suicide among the rural elderly in China (*n* = 484)

	Completed suicide		
Variables	OR	95% CI	*p*
Employment			
Employed	Ref	ref	
Unemployed	2.344	1.233–4.457	0.009
Retired	1.923	0.492–7.666	0.354
Living alone			
No	Ref	Ref	
Yes	2.176	1.113–4.254	0.023
Subjective social support			
Higher group (>5)	Ref	Ref	
Lower group (⩽5)	2.185	1.243–3.843	0.007
Depressive symptoms			
Without (<10)	Ref	Ref	
With (⩾10)	6.700	3.405–13.182	<0.001
Hopelessness			
Lower group (⩽12)	Ref	Ref	
Higher group (>12)	7.253	3.764–13.974	<0.001
Higher hopelessness × higher loneliness	2.446	1.089–5.492	0.030

*Note*: Adjusted for marital status (stable or unstable), being left-behind (yes or no), social interaction (⩽5 or >5), instrumental social support (⩽8 or >8) and loneliness (⩽13 or >13). OR, odds ratio; CI, confidence interval.

## Discussion

In this PA study, the CHAID tree model showed that loneliness, hopelessness and depressive symptoms had close associations with completed suicide and that loneliness and hopelessness interacted with each other. The results of the multivariable regression model did not indicate the independent effect of loneliness on suicide. However, the interaction effect between hopelessness and loneliness was significant. In order words, older adults in rural China with both higher levels of hopelessness and loneliness were twice as likely to commit suicide. The interpersonal theory of suicide may explain this result (Van Orden *et al*., 2010), suggesting that loneliness, one facet of thwarted belongingness, is associated with an elevated suicidal risk, particularly when the feeling of loneliness is chronic or prolonged. However, loneliness (or thwarted belongingness) alone does not cause active suicidal desire, whereas its interaction with hopelessness and perceived burdensomeness does (Van Orden *et al*., 2010).

The first layer of the classification tree was hopelessness, which had the largest effect on completed suicide in the multivariable model (OR = 7.25, 95% CI: 3.76–13.97). Many studies have demonstrated the importance of hopelessness as a risk factor for suicidality (Beck *et al*., 1985; Miranda *et al*., 2012; O'Connor *et al*., 2013). A recent meta-analysis of longitudinal studies showed that hopelessness significantly predicted suicidal ideation [weighted mean odds ratio (wOR) = 2.19, 95% CI: 1.60–3.00], suicidal attempt (wOR = 1.95, 95% CI: 1.59–2.39) and suicide death (wOR = 1.98, 95% CI: 1.46–2.69) (Ribeiro *et al*., 2018). In line with worldwide risk-factor guidelines (World Health Organization, 2008, 2016), our findings indicate that the detection of elderly people at suicide risk in rural China depends on the regular evaluation of hopelessness in that population.

PA studies have repeatedly reported depression as the most common mental illness among suicide decedents (Conwell *et al*., 1996; Harwood *et al*., 2001; Phillips *et al*., 2002; Zhang and Zhou, 2009). We also found that depression was a major risk factor for suicide among the elderly in rural China. Depressive symptoms were more prevalent among elderly suicides than the controls (94.2 *v*. 38.8%). Depression comprised of the second layer of the classification tree, under the category of a lower level of hopelessness. This finding suggests that even among those with a lower level of hopelessness, depression was associated with an elevated risk of suicide. Specifically, older adults suffering from depressive symptoms were six times more likely to commit suicide than those without depressive symptoms (OR = 6.70, 95% CI: 3.41–13.18).

The association between social support and suicidal behaviours has received abundant attention in the literature. The results of the current study are consistent with previous findings, which indicate that perceived or subjective social support is a protected factor of suicidality (Turvey *et al*., 2002; Madeleine Mellqvist *et al*., 2012; Xu *et al*., 2016; Liu *et al*., 2018). In the current study, suicide cases had a significantly lower level of social support than the controls, and subjective support had an independent effect on suicide risk. Furthermore, lower social interaction and instrumental support did not remain in the multivariable model. A U.S. study also reported no association between instrumental support and suicidality (Raue *et al*., 2007). These findings suggest that some aspects of social support may mitigate the risk of suicidal behaviour, while others may not (Madeleine Mellqvist *et al*., 2012). The different dimensions of social support should be measured separately.

Living alone was more common in suicide cases than in controls (26.4 *v*. 14.5%). In the multivariate regression model, living alone was independently associated with increased suicide risk. This result parallels findings from another study on community-dwelling, older adults in rural China (Hu, 2016). Living alone is related to other risk factors for suicide, such as feelings of loneliness, a lack of social support and depression (Wang *et al*., 2017; Djundeva *et al*., 2019; Kim *et al*., 2018). The increase in the number of older adults feeling left behind or living alone in rural China (Cai, 2018; Zhou *et al*., 2019) warrants more attention to this population's psychological well-being.

Unemployment was more common in suicide cases, aligning with previous reports (Inoue *et al*., 2007; Bastia and Kar, 2009). In rural China, older adults who are unemployed may suffer from financial strain if they lack a steady income or retirement pension. However, the occurrence was also very high in the controls. Thus, unemployment may confer risk for suicide only among those who are vulnerable or only when it causes certain negative outcomes. According to the interpersonal theory of suicide, the distress caused by unemployment may result in perceived burdensomeness, which may, in turn, elevate the risk for suicide (Van Orden *et al*., 2010).

The current study has several limitations. Though PA is a widely used method to explore the risk factors of completed suicide, it has methodological limitations. Its most debated aspect is the validity of data provided by proxy informants, especially regarding intra-experiences or feelings like loneliness. However, we found that subject-proxy concordance for the ULS-6 was fair (ICC = 0.447) in the living controls, which supported the validity of proxy-based data on loneliness in older people in rural China (Niu *et al*., 2018).

In conclusion, we found that hopelessness was the most important risk factor for suicide among the elderly in rural China. Loneliness is associated with suicidality through hopelessness. Depressive symptoms, living alone, unemployment and lower levels of subjective social support were also associated with an elevated risk for suicide in this vulnerable population. Regular evaluation of loneliness, hopelessness and depression can help detect elderly people who are at suicide risk. Interventions targeting social support systems (e.g. increasing social connections and enhancing intimate relationships), particularly among older adults living alone, are needed to alleviate this population's feelings of loneliness and hopelessness. Treating depression is also key to preventing suicide among elderly people in rural China.

## Data

All relevant data are within the paper. The datasets used or analysed during the current study are available from the corresponding author on reasonable request.
